# Inhibition of autophagy antagonizes breast cancer brain metastogenesis and augments the anticancer activity of lapatinib

**DOI:** 10.1002/ctm2.1662

**Published:** 2024-04-24

**Authors:** Steffan T. Nawrocki, Claudia M. Espitia, Maria Janina Carrera Espinoza, Trace M. Jones, Madison E. Gamble, Sruthi Sureshkumar, Mengyang Chang, Wei Wang, Jennifer S. Carew

**Affiliations:** ^1^ Department of Medicine, Division of Hematology and Oncology University of Arizona Cancer Center Tucson Arizona USA; ^2^ Department of Urology University of Arizona Tucson Arizona USA; ^3^ Department of Chemistry and Biochemistry University of Arizona Tucson Arizona USA; ^4^ Department of Pharmacology and Toxicology University of Arizona Tucson Arizona USA; ^5^ Arizona Center for Drug Discovery University of Arizona Tucson Arizona USA

Dear Editor,

Brain metastases are the most prevalent adult central nervous system tumours with 20%−30% of cases resulting from breast cancer patients, particularly those with triple‐negative (ER‐, PR‐, HER2 unamplified) and HER2 amplified disease. The prognosis for individuals with brain metastases from breast cancer continues to be extremely unfavourable, with a mere 20% achieving 5‐year survival.^1^ New targeted approaches are clearly needed to improve outcomes for this patient population. Autophagy is a lysosomal catabolic process that controls the turnover of organelles and selected proteins.^2^, ^3^ During metastogenesis, tumour cells face diverse stressors including nutrient and oxygen deprivation and may activate autophagy to avert bioenergetic failure and sustain their survival.^4^ We investigated the role of autophagy competence as a regulator of the brain metastogenic potential of breast cancer cells and assessed inhibition of autophagy as a clinically actionable approach to prevent and treat brain metastases.

We first used the well characterized breast cancer cell line MDA‐MB‐231 and its brain‐targeted metastatic variants (231‐BR and 231‐BR‐HER2) to investigate differences in energy metabolism in cells that preferentially metastasize to the brain (Figure S1A,B).^5^, ^6^, ^7^ Global metabolic profiling identified the nucleotide biosynthesis super pathway as one of the most significantly altered between parental and brain metastatic breast cancer cells (Figure 1A,B). Quantification of individual nucleotide pathway components revealed that adenosine 5′‐monophosphate (AMP) and adenosine 5′‐disphosphate (ADP_ levels were remarkably higher, while ATP levels were significantly lower in the brain metastatic variants (Figure 1C). Notably, differences in ATP production between other types of primary tumours and metastases were also recently reported.^8^ We detected diminished levels of the TCA cycle intermediates citrate, cis‐aconitate, and malate (Figure S2), lower pyrophosphate and higher phosphate levels (Figure S3), lower ATP levels (Figure 1D) and significantly higher levels of FAD+ and NADH in brain metastatic cells (Figure S4).

**FIGURE 1:**
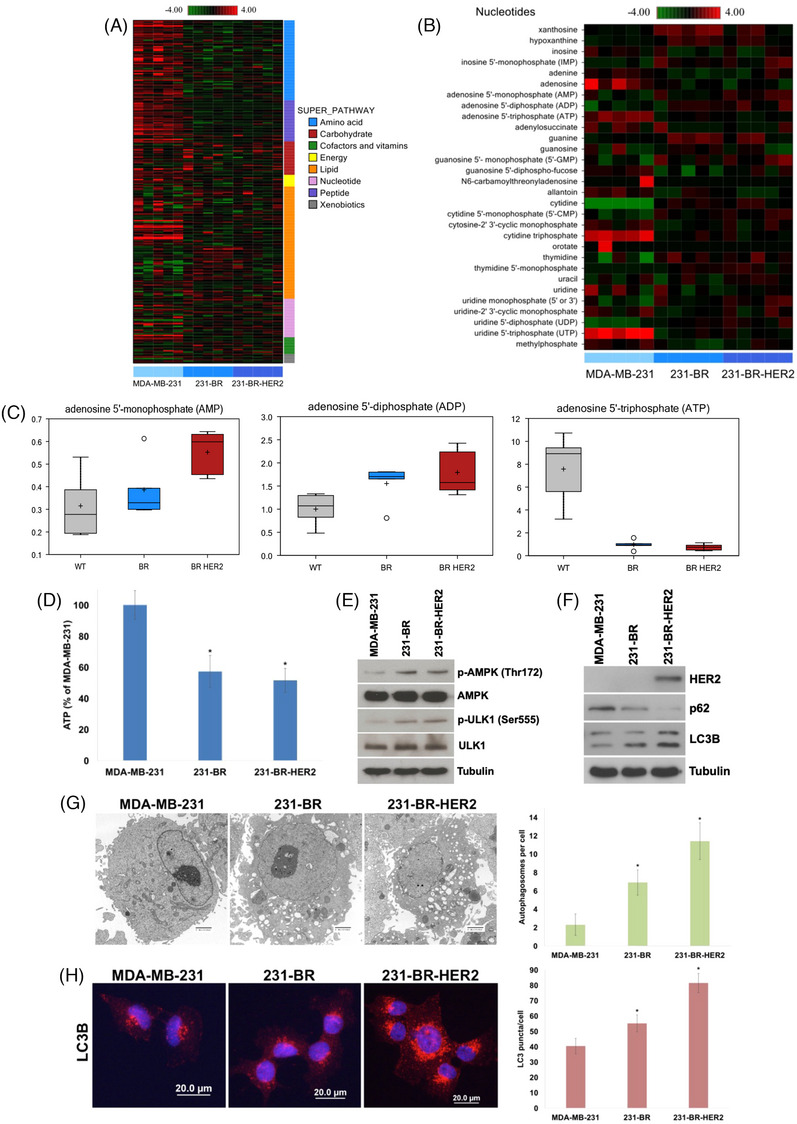
Brain metastatic 231‐BR and 231‐BR‐HER2 cells display altered metabolic pathways characterized by increased AMP, low ATP, and upregulated autophagy. (A) A total of 500 000 of parental MDA‐MB‐231, 231‐BR and 231‐BR‐HER2 cells were plated and harvested after 24 h. Cells were processed for global metabolic analyses as described in the Materials and Methods in the Supporting Information. Heatmap depicts super pathway analysis, *n* = 5 for each experimental condition. (B) Cells were processed for global metabolic analyses as described in the Materials and Methods in the Supporting Information. Heatmap specifically depicts nucleotide metabolism pathway analysis, *n* = 5 for each experimental condition. (C) Quantification of AMP, ADP and ATP by global metabolomic analysis. Box plots depict the levels of each individual component, *n* = 5 ± standard deviation (SD). (D) Measurement of ATP levels in the brain metastatic variants. A total of 500 000 cells were plated of parental and each variant. ATP levels were determined by ATPlite assay. Mean ± SD, *n* = 8. ^*^Indicates a significant difference from parental MDA‐MB‐231 cells, *p* < 0.05. (E) Parental and brain metastatic variants were plated for 24 h. Cells were collected and the levels of p‐AMPK, AMPK, p‐ULK1, ULK1 and beta‐tubulin were determined by immunoblotting. (F) Cells were plated and harvested 24 h later. The levels of HER2, p62, and LC3B were determined by immunoblotting. Tubulin was used as a loading control. (G) The number of autophagosomes per cell were measured by transmission electron microscopy (TEM) and counted manually. Representative images are shown. Mean ± SD, *n* = 10. Scale bar = 2 μm. ^*^Represents a significant difference from MDA‐MB‐231 parental cells, *p* < 0.05. (H) The levels of LC3 puncta per cell were measured by immunocytochemistry and quantified using ImageJ software. Representative images are shown. Mean ± SD, *n* = 10. Scale bar = 20 μm. ^*^Denotes a significant difference from parental MDA‐MB‐231 cells.

To determine if brain metastatic cells utilize autophagy to compensate for their altered metabolic profiles, we assessed the levels of phospho‐AMPK and its downstream target phospho‐ULK1. Both were elevated in the brain‐targeted variants (Figure 1E). Additionally, brain metastatic cells exhibited decreased p62 levels and increased LC3B expression (Figure 1F), and increased numbers of autophagosomes (Figure 1G) and LC3B puncta (Figure 1H), which collectively support enhanced basal autophagy. We next genetically impaired the essential autophagy gene *ATG7* in 231‐BR‐HER2 cells using shRNA to investigate the role of autophagy during brain metastogenesis in mice (Figure 2A). Representative brains were collected from mice on day 23 and whole brain fluorescence imaging was performed to quantify brain metastases. Metastatic burden was dramatically reduced in mouse brains injected with cells with stably silenced *ATG7* (Figure 2B). To evaluate how autophagy competence impacts the number and size of breast cancer brain metastases, brains from each group were serially cut and stained with H&E and subjected to image‐based quantification. Notably, the number of both micrometastases (<50 μm^2^) and large metastases (>50 μm^2^) were significantly reduced in the *ATG7* shRNA injected mice (Figures 2C and S5). The remaining mice were monitored over 100 days to verify the long‐term effect of *ATG7* silencing on autophagic inhibition and animal survival. Consistent with their decreased brain metastatic burden, mice injected with 231‐BR‐HER2 *ATG7* shRNA cells displayed elevated p62 levels (Figure 2D) and experienced considerably increased overall survival (Figure 2E).

**FIGURE 2:**
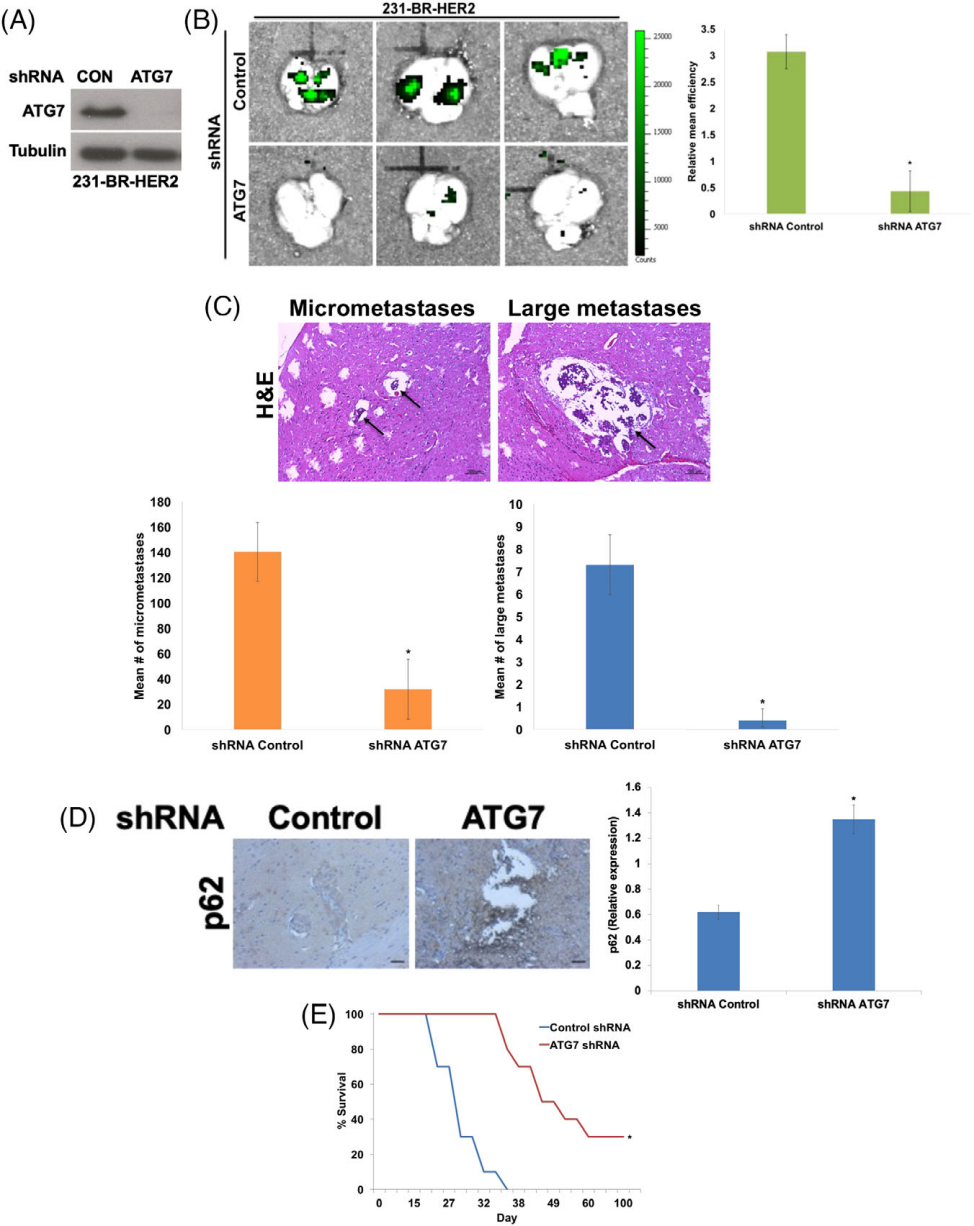
Autophagy contributes to breast cancer brain metastases. (A) Lentiviral shRNA knockdown of ATG7. 231‐BR‐HER2 cells were transfected with lentiviral shRNA particles targeting ATG7 or a nontargeted control. The levels of ATG7 were determined by immunoblotting. (B) Silencing of ATG7 significantly reduces brain metastasis. Nontarget control shRNA or ATG7 shRNA 231‐BR‐HER2‐eGFP cells were intracardially injected to establish brain metastases. Mice were monitored and brains were collected from each group on day 23. Brain metastases were determined by whole brain fluorescent imaging using the IVIS Xenogen imaging system. Fluorescence was quantified using Living Image software. Mean ± standard deviation (SD), *n* = 3. ^*^Indicates a significant difference from nontarget control shRNA injected cells, *p* < 0.05. (C) Quantification of brain micrometastases and large metastases in nontarget control and ATG7 shRNA 231‐BR‐HER2 intracardially injected mice. Brains were harvested and serially sectioned for H&E staining. The numbers of micrometastases and large metastases were quantified by light microscopy and manual counting. Mean ± SD, *n* = 10. ^*^Denotes a significant difference compared to the control shRNA group, *p* < 0.05. (D) Quantification of p62 immunohistochemistry. Levels of p62 were measured by immunohistochemistry in brain sections from nontarget control and ATG7 shRNA tumours. Relative expression was quantified using ImageJ software. Scale bar = 500 μm. *N* = 10 +/− SD. ^*^Indicates a significant difference compared to the control shRNA group, *p* < 0.05. (E) Nontarget control or ATG7 shRNA 231‐BR‐HER2 cells were intracardially injected into mice to establish brain metastases. Mice were monitored until day 100 of the experiment. Overall survival was determined by Kaplan–Meier survival analysis, *n* = 10 per group. ^*^Denotes a significant difference from nontarget control shRNA group, *p* < 0.05.

The factors that limit the efficacy of the FDA approved HER2/EGFR inhibitor lapatinib against breast cancer brain metastases have yet to be fully elucidated, but its effects on HER2/EGFR/AKT suggested that autophagy may play a vital role (Figure 3A).^9^, ^10^ Additional experiments showed that lapatinib treatment further induced autophagy as measured by increased LC3B levels, decreased p62 expression, stimulation of autophagic flux, and increased autophagosomes and LC3B puncta (Figure 3A–D).

**FIGURE 3:**
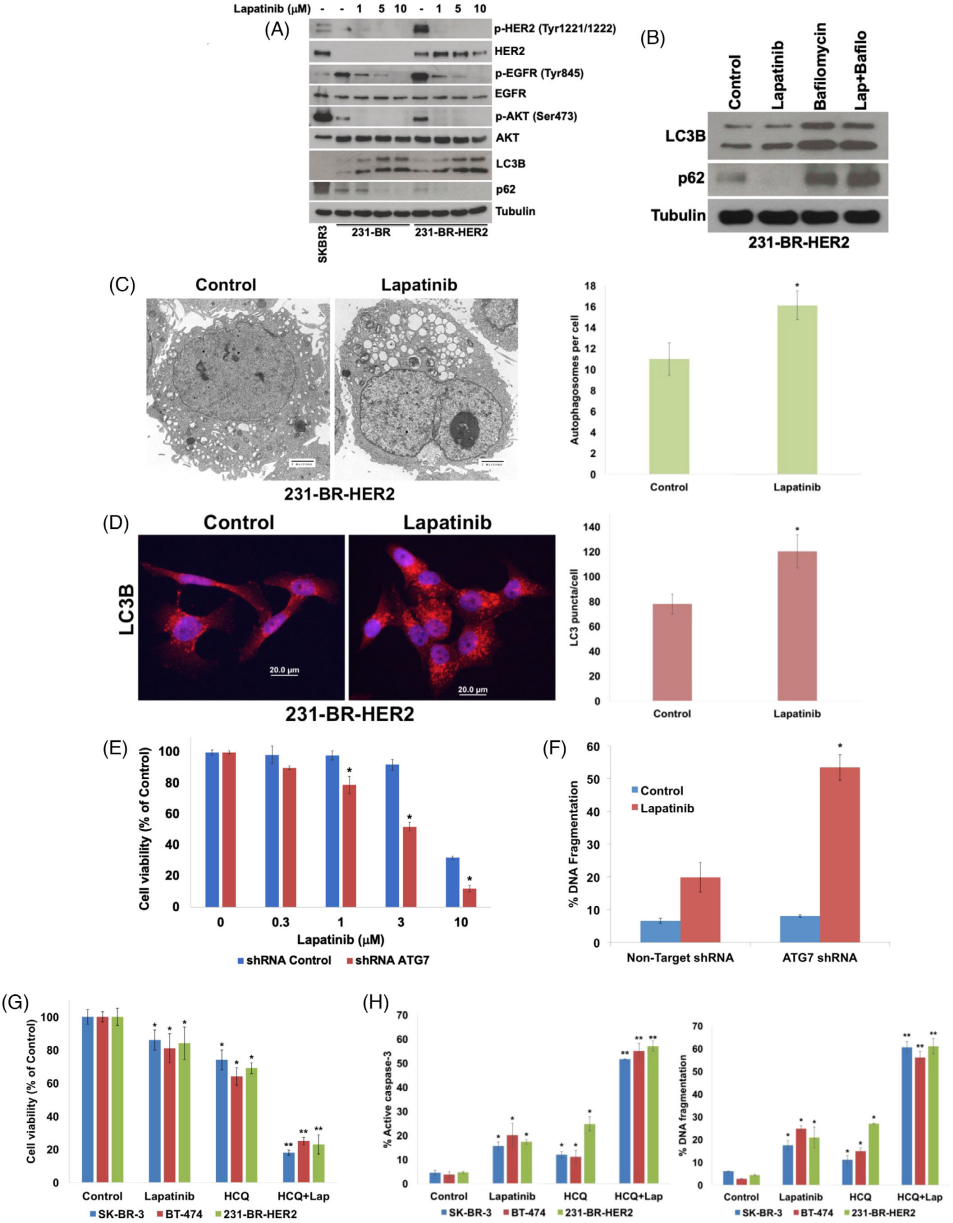
Inhibition of autophagy enhances the anticancer activity of lapatinib. (A) Lapatinib decreases the expression of p‐HER2, p‐EGFR, p‐AKT and p62 while increasing LC3B levels. 231‐BR‐HER2 cells were treated with the indicated concentrations of lapatinib for 24 h. SK‐BR‐3 cells served as a positive control p‐HER2, P‐EGFR, and p‐AKT. Protein expression was determined by immunoblotting. (B) Bafilomycin A1 blocks lapatinib‐induced reduction in p62 expression. 231‐BR‐HER2 cells were treated with 3 μM lapatinib, 100 nM bafilomycin A1, or both agents for 48 h. LC3B and p62 expressions were determined by immunoblotting. (C). Lapatinib induces autophagy. 231‐BR‐HER2 cells were treated with 3 μM lapatinib for 24 h. The number of autophagosomes per cell were measured by TEM and counted manually. Representative images are shown. Mean ± standard deviation (SD), *n* = 10. Scale bar = 2 μm. ^*^Represents a significant difference from control, *p* < 0.05. (D) Lapatinib treatment increases LC3B puncta. 231‐BR‐HER2 cells were treated with 3 μM lapatinib for 24 h. The levels of LC3 puncta per cell were measured by immunocytochemistry and quantified using ImageJ software. Representative images are shown. Mean ± SD, *n* = 10. Scale bar = 20 μm. ^*^Denotes a significant difference from control. (E) Silencing of ATG7 augments the activity of lapatinib. ATG7 expression in 231‐BR‐HER2 cells was knocked down as shown in Figure 2A. Nontarget control and ATG7 shRNA cells were treated with the indicated concentrations of lapatinib for 72 h. Cell viability was measured by MTT assay. Mean ± SD, *n* = 4. ^*^Represents a significant difference from nontarget shRNA control cells treated with the same concentration of lapatinib, *p* < 0.05. (F) Silencing ATG7 enhances lapatinib‐induced apoptosis. Nontarget control and ATG7 shRNA cells were treated with 3 μM lapatinib for 48 h. DNA fragmentation was measured by PI‐FACS analysis. Mean ± SD, *n* = 3. ^*^Indicates a significant difference from nontarget shRNA control cells treated with the same concentration of lapatinib, *p* < 0.05. (G) HCQ enhances the anticancer activity of lapatinib in HER2+ breast cancer models. Cells were treated with 25 μM HCQ, 3 μM lapatinib (for 231‐BR‐HER2) or 30 nM lapatinib (for SK‐BR‐3 and BT‐474), and the combination for 72 h. Cell viability was determined by MTT assay. Mean ± SD, *n* = 4. ^*^Indicates a significant difference from control and ^**^represents a significant difference from both HCQ and lapatinib monotherapy, *p* < 0.05. (H) HCQ significantly augments lapatinib‐induced apoptosis. Cells were treated with 25 μM HCQ, 3 μM lapatinib (for 231‐BR‐HER2) or 30 nM lapatinib (for SK‐BR‐3 and BT‐474), and the combination for 48 h. Apoptosis was measured by active caspase‐3 assay (left) and PI‐FACS analysis (right). Mean ± SD, *n* = 3. ^*^Represents a significant difference from control and ^**^indicates a significant difference from either monotherapy, *p* < 0.05.

We next assessed whether disrupting autophagy improved the anticancer activity of lapatinib. *ATG7* knockdown significantly enhanced the ability of lapatinib to decrease 231‐BR‐HER2 cell viability (Figure 3E) and considerably increased lapatinib‐mediated apoptosis (Figure 3F). Pharmacological autophagy inhibition with hydroxychloroquine (HCQ) also augmented lapatinib activity in 231‐BR‐HER2 cells and the HER2+ cell lines SK‐BR‐3 and BT‐474 (Figure 3G,H). Finally, we investigated whether autophagy inhibition is an effective strategy to improve the activity of lapatinib against breast cancer brain metastasis. Five days following intracardiac injection of 231‐BR‐HER2‐eGFP cells, mice were randomized into vehicle, lapatinib, HCQ, or combination treatment groups. On day 23, brains from each group were harvested from mice and fluorescently imaged. Both lapatinib and HCQ monotherapies induced a significant, but modest decrease in brain metastases (Figure 4A). Importantly, combination treatment was significantly more effective in reducing the burden of brain metastases compared to either single agent (Figure 4A). Evaluation of brain sections showed that combination treatment significantly decreased the number of both micrometastases and large metastases (Figure 4B). HCQ treatment also inhibited lapatinib‐induced degradation of p62 indicating autophagic inhibition (Figure 4C). Importantly, combination treatment significantly extended animal survival compared to either monotherapy (Figure 4D). Collectively, our results show that genetic or pharmacological inhibition of autophagy promotes considerably enhanced sensitivity to lapatinib, which results in increased duration of overall survival of 231‐BR‐HER2 brain metastasis‐bearing animals (Figure 4E).

**FIGURE 4:**
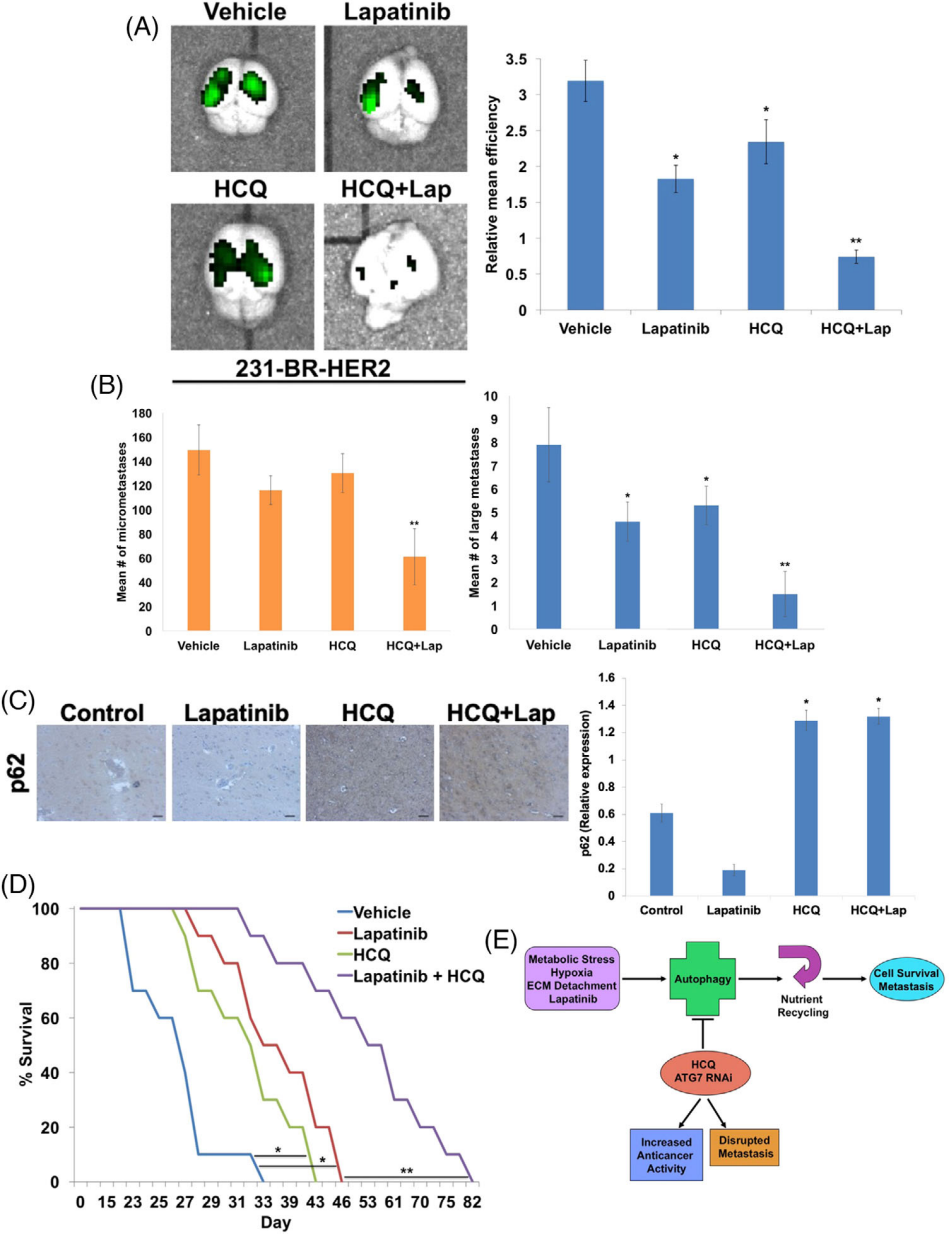
HCQ cooperates with lapatinib to reduce brain metastases and increase animal survival. (A) 231‐BR‐HER2‐eGFP cells were intracardially injected into mice to establish brain metastases. After 5 days, mice were treated with 100 mg/kg lapatinib orally, 60 mg/kg HCQ IP, or both agents daily. On day 23, the brains from representative animals were imaged using the IVIS Xenogen imaging system to quantify eGFP expressing 231‐BR‐HER2 metastases. Fluorescence was quantified using Living Image software. Mean ± standard deviation (SD), *n* = 3. ^*^Indicates a significant difference from vehicle control and ^**^represents a significant difference from either single agent treatment, *p* < 0.05. (B) Quantification of brain micrometastases and large brain metastases in lapatinib, HCQ, and combination treated mice. Brains were harvested and serially sectioned for H&E staining. The numbers of micrometastases and large metastases were quantified by light microscopy and manual counting. Mean ± SD, *n* = 10. ^*^Denotes a significant difference compared to vehicle control and ^**^compared to either monotherapy, *p* < 0.05. (C) Quantification of p62 levels by immunohistochemistry. Levels of p62 were measured by immunohistochemistry in 231‐BR‐HER2 brain metastases treated with lapatinib, HCQ and the combination. Relative expression was quantified using ImageJ software. Scale bar = 500 μm. *N* = 10 +/− SD, *p* < 0.05. ^*^Denotes a significant increase from control and lapatinib groups. (D) 231‐BR‐HER2 cells were intracardially injected into mice to establish brain metastases. After 5 days, mice were treated with 100 mg/kg lapatinib orally, 60 mg/kg HCQ IP, or both agents daily. Mice were monitored daily for any signs of toxicity. Overall survival was determined by Kaplan–Meier survival analysis, *n* = 10 per group. ^*^Indicates a significant difference from vehicle control and ^**^represents a significant difference from either monotherapy, *p* < 0.05. (E) Graphical illustration depicting the current model that inhibition of autophagy decreases breast cancer brain metastasis and augments the anticancer activity of lapatinib.

The management of breast cancer metastases in the brain remains a formidable challenge and patients with these lesions have a dismal prognosis. We leveraged metabolomic analyses to uncover a critical alteration in the energy production of brain‐seeking metastatic breast cancer cells that results in enhanced autophagy. Silencing of the essential autophagy gene *ATG7* significantly decreased the formation and number of brain metastases leading to increased animal survival. Importantly, pharmacologically targeting autophagy with HCQ significantly increased the anticancer activity of lapatinib, reduced the number of brain metastases, and extended animal survival. Our research establishes rationale for clinically assessing this therapeutic approach to treat breast cancer brain metastases and enhance lapatinib treatment. Other areas for future investigation include: (1) evaluating the role of autophagy in regulating metastasis to other organ sites; (2) determining the benefit of autophagy inhibition with respect to the efficacy of other breast cancer therapeutics that cross the blood brain barrier; and (3) the development of more potent targeted autophagy inhibitors.

## AUTHOR CONTRIBUTIONS

Steffan T. Nawrocki designed the study, performed research and contributed to data analysis and manuscript preparation; Claudia M. Espitia performed research, and contributed to data analysis and manuscript preparation; Maria Janina Carrera Espinoza contributed to data analysis and manuscript preparation; Trace M. Jones performed research and contributed to data analysis; Madison E. Gamble contributed to data analysis and manuscript preparation; Sruthi Sureshkumar contributed to data analysis and manuscript preparation; Mengyang Chang contributed to data analysis and manuscript preparation; Wei Wang contributed to data analysis and manuscript preparation; and Jennifer S. Carew designed the study, performed research and contributed to data analysis and manuscript preparation.

## CONFLICT OF INTEREST STATEMENT

Jennifer S. Carew, Steffan T. Nawrocki and Wei Wang are co‐founders of Majestic Therapeutics, LLC. The other authors declare no conflicts of interest.

## ETHICS STATEMENT

The animal studies were approved by the Institutional Animal Care and Use Committee under protocol 16‐094.

## Supporting information

Supporting Information

Supporting Information

Supporting Information

Supporting Information

Supporting Information

Supporting Information

## References

[1] Patel RR , Mehta MP . Targeted therapy for brain metastases: improving the therapeutic ratio. Clin Cancer Res. 2007;13(6):1675‐1683. doi:10.1158/1078-0432.CCR-06-2489 17363520

[2] Jones TM , Carew JS , Nawrocki ST . Therapeutic targeting of autophagy for renal cell carcinoma therapy. Cancers. 2020;12(5):1185. doi:10.3390/cancers12051185 32392870 PMC7281213

[3] Nawrocki ST , Han Y , Visconte V , et al. The novel autophagy inhibitor ROC‐325 augments the antileukemic activity of azacitidine. Leukemia. 2019;33(12):2971‐2974. doi:10.1038/s41375-019-0529-2 31358855 PMC7462348

[4] Degenhardt K , Mathew R , Beaudoin B , et al. Autophagy promotes tumor cell survival and restricts necrosis, inflammation, and tumorigenesis. Cancer Cell. 2006;10(1):51‐64. doi:10.1016/j.ccr.2006.06.001 16843265 PMC2857533

[5] Gril B , Palmieri D , Bronder JL , et al. Effect of lapatinib on the outgrowth of metastatic breast cancer cells to the brain. J Natl Cancer Inst. 2008;100(15):1092‐1103. doi:10.1093/jnci/djn216 18664652 PMC2575427

[6] Liu H , Kato Y , Erzinger SA , et al. The role of MMP‐1 in breast cancer growth and metastasis to the brain in a xenograft model. BMC Cancer. 2012;12:583. doi:10.1186/1471-2407-12-583 23217186 PMC3526403

[7] Palmieri D , Bronder JL , Herring JM , et al. Her‐2 overexpression increases the metastatic outgrowth of breast cancer cells in the brain. Cancer Res. 2007;67(9):4190‐4198. doi:10.1158/0008-5472.CAN-06-3316 17483330

[8] Bartman CR , Weilandt DR , Shen Y , et al. Slow TCA flux and ATP production in primary solid tumours but not metastases. Nature. 2023;614(7947):349‐357. doi:10.1038/s41586-022-05661-6 36725930 PMC10288502

[9] Lin NU , Dieras V , Paul D , et al. Multicenter phase II study of lapatinib in patients with brain metastases from HER2‐positive breast cancer. Clin Cancer Res. 2009;15(4):1452‐1459. doi:10.1158/1078-0432.CCR-08-1080 19228746

[10] Lin NU , Guo H , Yap JT , et al. Phase II study of lapatinib in combination with trastuzumab in patients with human epidermal growth factor receptor 2‐positive metastatic breast cancer: clinical outcomes and predictive value of early [18F]fluorodeoxyglucose positron emission tomography imaging (TBCRC 003). J Clin Oncol. 2015;33(24):2623‐2631. doi:10.1200/JCO.2014.60.0353 26169615 PMC4534525

