# Supplementary material for: Inhibition of autophagy antagonizes breast cancer brain metastogenesis and augments the anticancer activity of lapatinib
Source: Clin Transl Med. 2024 Apr 24;14(4):e1662. doi: 10.1002/ctm2.1662 (PMC11043092; doi:10.1002/ctm2.1662)
Supplement: Supplementary file 1 — Supporting Information [file CTM2-14-e1662-s003.docx]

**Supplemental Materials and Methods**

**Cells and cell culture**

Breast cancer cell lines SK-BR-3, MDA-MB-231, and BT-474 were obtained from American Type Culture Collection (ATCC, Manassas, VA). The MDA-MB-231 “brain seeking” breast cancer cell line expressing enhanced green fluorescent protein (eGFP) and transfected with HER2 (231-BR-HER2) (pCMV4.HER2 full length human cDNA) and vector control (231-BR) were kindly provided by Dr. Patricia Steeg (National Institutes of Health, Bethesda, MD). Cells were authenticated using short tandem repeat DNA profiling procedures. Cells were cultured in RPMI-1640 medium containing 0.750 mg/ml zeocin enriched with 10% FBS in 5% CO_2_ at 37 °C. Following a PBS wash, cells were harvested and incubated with 0.25% trypsin, 1X sodium bicarbonate solution, and 2.21 mM EDTA for 5 min at 37 °C. A Beckman Coulter Vi-CELL XR Cell Viability Analyzer (Beckman-Coulter, Brea, CA) was used to count the cells.

**Chemicals and antibodies**

Bafilomycin A1, 3-(4,5-dimethylthiazol-2-yl)-2,5-diphenyltetrazolium bromide (MTT), and propidium iodide (PI) were purchased from Sigma (St. Louis, MO). HCQ and lapatinib were acquired from SelleckChem (Houston, TX). The antibodies anti-phospho-AMPK (Thr172) (#2535), AMPK (#2532), HER2 (#2242), LC3B (#3868), ATG7 (#8558), phospho-HER2 (Tyr1221/1222) (#2243), phospho-EGFR (Tyr845) (#6963), EGFR (#2085), phospho-AKT (Ser473) (#4058), ULK1 (#8054), phospho-ULK1 (Ser555) (#5869), and AKT (#9272) were obtained from Cell Signaling (Danvers, MA). Anti-β-Tubulin (#T7816) antibody was purchased from Sigma. Anti-SQSTM1/p62 (#ab91526) was acquired from Abcam (Cambridge, MA). Goat anti-rabbit Alexa-Fluor 594, goat anti-mouse Alexa-Fluor 488, and Prolong Gold antifade with DAPI was purchased from ThermoFisher (Waltham, MA). Goat anti-rabbit or anti-mouse HRP tagged secondary antibodies were obtained from Jackson ImmunoResearch Laboratories (West Grove, PA).

**Sample preparation and metabolic profiling**

Our untargeted metabolic profiling strategy harnessed three distinct platforms: gas chromatography/mass spectrometry (GC/MS), ultra-high performance liquid chromatography/tandem mass spectrometry (UHLC/MS/MS^2^) optimized for basic species, and acidic species. In brief, cells were homogenized in a minimum water volume and 100 μL was withdrawn for subsequent analyses. To assess extraction efficiency, protein was precipitated from the homogenized cells using methanol containing four standards using an automated liquid handler (Hamilton LabStar, Salt Lake City, UT). The obtained supernatant was split into equal aliquots for analysis on the three platforms. Aliquots that were dried under nitrogen and vacuum-desiccated were consequently reconstituted in 50 μL 6.5 mM ammonium bicarbonate in water or in 50 μL 0.1% formic acid in water, pH 8 for the two UHLC/MS/MS^2^ analyses or derivatized to a final volume of 50 μL for GC/MS analysis using equal parts bistrimethyl-silyl-trifluoroacetamide and solvent mixture acetonitrile:dichloromethane:cyclohexane (5:4:1) with 5% triethylamine for 1 h at 60 °C. In conjunction with the experimental samples, we analyzed three types of controls to ensure the robustness of our analytical approach. Technical replicates were generated from pooled experimental samples and were employed consistently throughout the dataset. Samples of extracted water were used as process blanks. Additionally, to monitor instrument performance a cocktail of standards was spiked into each analyzed sample. To accomplish the UHLC/MS/MS^2^ analysis, aliquots were divided using a Waters Acquity UPLC (Waters, Millford, MA) and examined using an LTQ mass spectrometer (ThermoFisher Scientific, Inc., Waltham, MA) comprised of a linear ion-trap (LIT) mass analyzer and an electrospray ionization (ESI) source. The MS device scanned 99–1000 m/z and alternated between MS^2^ and MS scans using dynamic exclusion with relatively 6 scans per second. Derivatized samples for GC/MS were divided on a 5% phenyldimethyl silicone column with a temperature ramp from 60 to 340 °C with helium as the carrier gas. Then the samples were evaluated on a Thermo-Finnigan Trace DSQ MS (ThermoFisher Scientific, Inc.).

**Metabolite identification and data analysis**

To identify metabolites, we used an automated method by comparing the ion properties of the experimental samples to a reference library of chemical standard entries. This library included crucial parameters such as molecular weight (m/z), associated MS spectra, preferred adducts, retention time, and in-source fragments. Additionally, a thorough quality control step was conducted through manual curation of the results. This entire process was facilitated using custom software developed at Metabolon. To ensure robustness and eliminate bias, both experimental and control samples were randomized across a one-day platform run. In cases where metabolite values were missing, these were presumed to be below the limits of detection. For subsequent statistical analyses and data presentation, these missing values were imputed using the compound minimum value imputation method. This imputation was achieved by the Bradford assay that was completed on the cell homogenate after normalizing the data to the total protein content of each sample. We performed statistical analysis on the log-transformed data using two software tools: Array Studio software (Omicsoft, Inc) and R (http://cran.r-project.org/), an open-source software package freely available to the scientific community. Additionally, Welch's t-tests were achieved to compare data between experimental groups. To account for multiple comparisons, the false discovery rate (FDR) was projected using q-values to assure the reliability and integrity of our statistical findings.

**Cell viability assay**

Cell viability was determined using MTT assay, which quantifies the conversion of MTT to formazan by the mitochondria of live cells. Briefly, 10,000 cells per well were seeded into 96-well plates and incubated for 24 h. Thereafter, cells were treated with lapatinib, HCQ, and the combination for 72 hours. MTT solution was then added and incubated at 37 °C under 5% CO_2_ for 2 h. Formazan absorbance was quantified at 570 nm using a Molecular Devices microplate reader. The formazan optical density of each experimental condition was normalized to the density of control cells to determine the estimated cell viability.

**Measurement of ATP**

Equal number of cells were plated. ATP levels were determined using the ATPlite assay kit, which measures the reaction of ATP with luciferase and D-luciferin. The assay was performed by following the manufacturer’s guidelines (Perkin Elmer, Waltham, MA).

**Immunoblotting**

Breast cancer cells were incubated with lapatinib, HCQ, or the combination of both drugs for 24 h as indicated. Afterwards, cells were lysed on ice in Triton X-100 lysis buffer (1% triton X-100, 150 mM NaCl, 25 mM Tris pH 7.5) with protease inhibitors for 1 h. Moreover, proteins were separated using SDS-PAGE and transferred to nitrocellulose membranes. Blots were probed with the designated antibodies and bands were identified by enhanced chemiluminescence (Alpha Innotech, San Leandro, CA). β-tubulin was used as a control to ensure equivalent loading.

**Transmission electron microscopy**

Transmission electron microscopy (TEM) of cells was conducted to visualize autophagosomes. In brief, breast cancer cells were untreated or treated with lapatinib for 24 h and harvested for imaging. Sections were cut in an LKB Ultracut microtome (Leica, Deer Park, IL), stained with uranyl acetate and lead citrate, and visualized using a JEM 1230 transmission electron microscope (JEOL, USA, Inc.). Images were obtained using the AMT Imaging System (Advanced Microscopy Techniques Corp, Woburn, MA). Manual counting was used to calculate the number of autophagosomes per cell.

**Quantitative RT-PCR (qRT-PCR)**

RNA was isolated from breast cancer cells using the RNeasy mini kit (Qiagen, Germantown, MD) according to the manufacturer’s guidelines. The high-capacity cDNA reverse transcription kit was used following the manufacturer’s (ThermoFisher, Waltham, MA) protocol to generate cDNA. Taqman primers for *HER2*, *MMP1,* and *GAPDH* were purchased from ThermoFisher. All experiments were carried out in triplicate.

**Apoptosis assays**

The active caspase-3 assay was performed after incubating the cells for 48 h with specific concentrations of the studied drugs. The assay was carried out using the FITC Active Caspase-3 Apoptosis Kit following the manufacturer’s protocol (BD BioSciences, Franklin Lakes, NJ). Quantification of FITC fluorescence intensity was accomplished on a BD FACSCelesta flow cytometer (BD BioSciences). All experiments were repeated three times.

Propidium Iodide (PI) staining was achieved to quantify DNA fragmentation. In brief, cells were incubated for 48 h using the specified drug concentrations. Following drug treatment, cells were stained for 2 h using 25 μg/mL PI, 0.1% Triton X-100, 0.1% sodium citrate solution at 4 °C. The sub-G0/G1 DNA content of the cells was assessed using a BD FACSCelesta flow cytometer.

**Immunocytochemistry**

Cells were plated in 2-well chamber slides for 24 h, washed with PBS, and fixed in 4% formaldehyde at room temperature for 15 min. Then, cells were permeabilized with 0.2% Triton X-100 for 10 min and incubated with an anti-LC3B primary antibody at 4 °C for 12 h. Next, a goat anti-rabbit AlexaFluor-594 secondary antibody was applied to visualize LC3B, while DAPI was utilized as a nuclear counterstain. Images were taken using a Zeiss Axio Vert.A1 microscope (Cambridge, UK). ImageJ software (NIH, Bethesda, MD) was used to measure the number of LC3B puncta/cell (n = 10).

**Immunohistochemistry**

Paraffin-embedded tumor sections were deparaffinized in xylene, exposed to a graded series of alcohol, and rehydrated in PBS (pH 7.5). Sections were microwaved in citrate buffer for five minutes to retrieve epitopes. Endogenous peroxide blocking was performed by incubation of samples in 3% H_2_O_2_ in methanol for 10 minutes. Samples were incubated in blocking solution (5% horse and 1% goat serum in PBS) for 20 minutes. Primary antibody targeting p62 was diluted in blocking solution. Samples were incubated with the primary antibody overnight at 4°C. After washing with PBS, samples were incubated with the appropriate secondary antibody for 60 minutes at room temperature. After washing with PBS, positive reactions were visualized using 3,3'-diaminobenzidine (Dako, Santa Clara, CA) for 15 minutes at room temperature. Slides were rinsed with water and counterstained for one minute with Gill’s hematoxylin (Sigma, St. Louis, MO). Images were captured on a Zeiss Axio Vert.A1 microscope with a 20X objective. ImageJ software with a Fiji plugin was used to quantify staining intensity (n = 10).

**shRNA silencing of *ATG7***

In accordance with the manufacturer’s guidelines (Santa Cruz Biotechnology, Santa Cruz, CA), 231-BR-HER2 cells were infected with lentiviral particles containing non-targeted (control) or target-specific short hairpin RNA (shRNA) directed at *ATG7.* Effective transfection was selected for with puromycin. Transfected cells were treated with the designated drugs and concentrations. Immunoblotting was used to determine knockdown efficiency.

***In vivo* evaluation**

The animal experiments were performed with the approval of the Institutional Animal Care and Use Committee of the University of Arizona (16-094) and were conducted in accordance with established animal welfare guidelines. Control shRNA or *ATG7* shRNA 231-BR-HER2-eGFP cells (1.75 × 10^5^ cells) were trypsinized, collected, washed in PBS, and injected into the left cardiac ventricle of female nude (nu/nu) mice. Mice were supervised for 100 days to determine animal survival. Mice were euthanized using CO_2_ asphyxiation if they exhibited symptoms of neurological impairment. To determine the presence of 231-BR-HER2-eGFP, an IVIS Xenogen imager (PerkinElmer, Waltham, MA) was used on Day 23 in random mouse brains excised from Control (n = 10) and *ATG7* (n = 10) shRNA animals. Fluorescence was quantified using Living Image software (PerkinElmer, Waltham, MA).

In addition to fluorescent imaging, mouse brains were fixed in 4% paraformaldehyde and prepared for paraffin embedding to determine the size and number of brain metastases. Brain sections were serially cut and stained with hematoxylin and eosin (H&E). For the experiment, 10 H&E serial sections were cut every 300 μm and the presence of metastases was visualized using a Zeiss Axio Vert.A1 microscope (Cambridge, UK). The presence of micrometastases (< 50 μm) and large metastases (> 50 μm) were counted.

For the therapeutic xenograft study, 231-BR-HER2-eGFP cells (1.75 × 10^5^ cells) were injected into the left cardiac ventricle of female nude mice to establish brain metastases. Treatment with lapatinib, HCQ, or both drugs was initiated 5 days after cell injection. Mice were randomized and treated with vehicle, 100 mg/kg lapatinib by oral gavage daily, 60 mg/kg HCQ IP daily, or both agents. Mice were monitored until the end of the study to determine animal survival. Mice were euthanized using CO_2_ asphyxiation if they displayed signs of neurological impairment. Random mouse brains were excised from each group (n = 10 per group) on Day 23 and imaged for the presence of 231-BR-HER2-eGFP using an IVIS Xenogen imager (PerkinElmer, Waltham, MA). Fluorescence was quantified using Living Image software (PerkinElmer, Waltham, MA). Following fluorescent imaging, the brains underwent the previously mentioned procedures of being fixed, paraffin embedded, serially sliced, H&E stained, and imaged. The presence of micrometastases (< 50 μm) and large metastases (> 50 μm) were determined by manual counting.

**Statistical Analyses**

Statistical significance of sample differences was assessed using the Student's *t* test, one-way ANOVA, and Kaplan-Meier analysis. At p < 0.05, differences were considered significant.
