# Supplementary figures and images for: Inhibition of autophagy antagonizes breast cancer brain metastogenesis and augments the anticancer activity of lapatinib
Source: Clin Transl Med. 2024 Apr 24;14(4):e1662. doi: 10.1002/ctm2.1662 (PMC11043092; doi:10.1002/ctm2.1662)

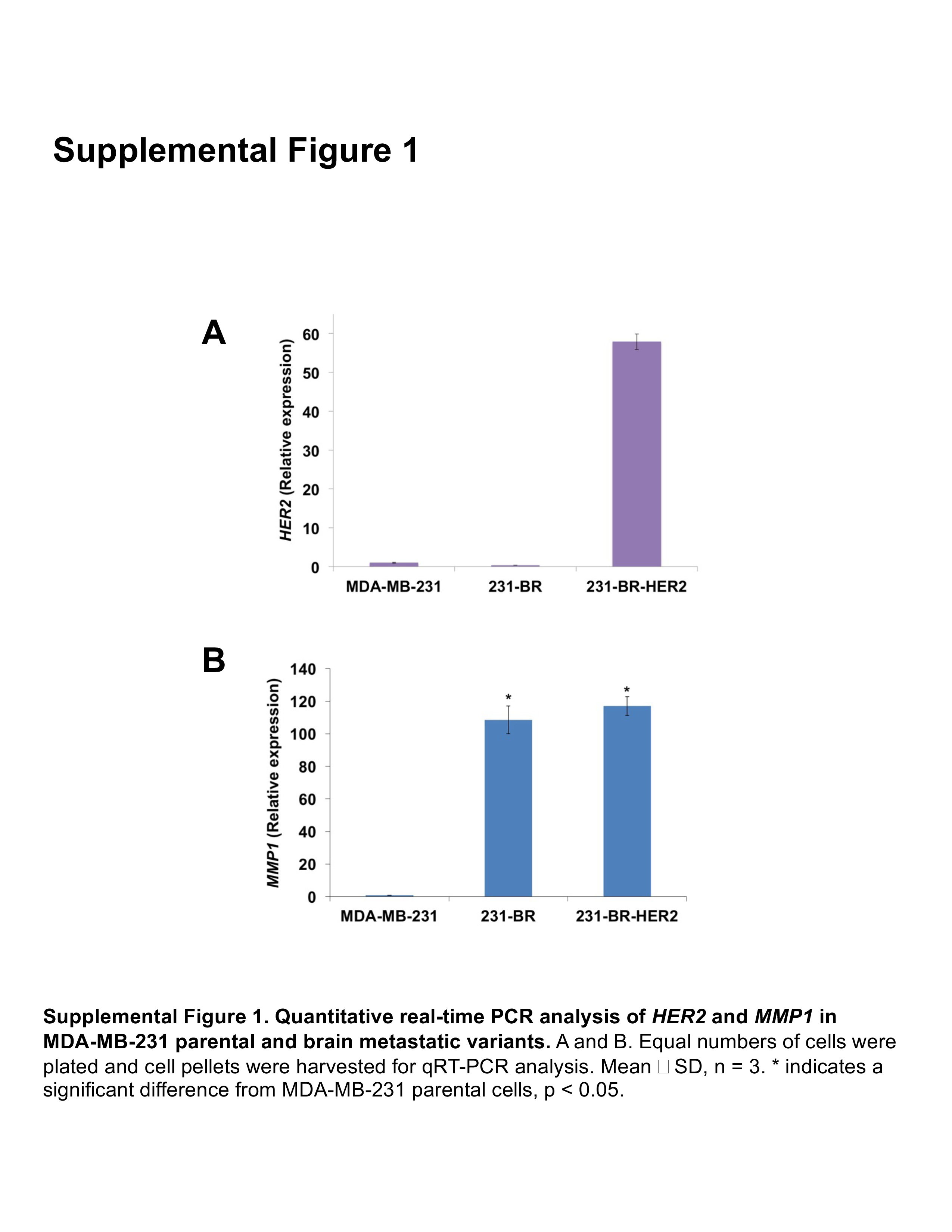

Supplement: Supplementary file 2 — Supporting Information [file CTM2-14-e1662-s006.jpg]

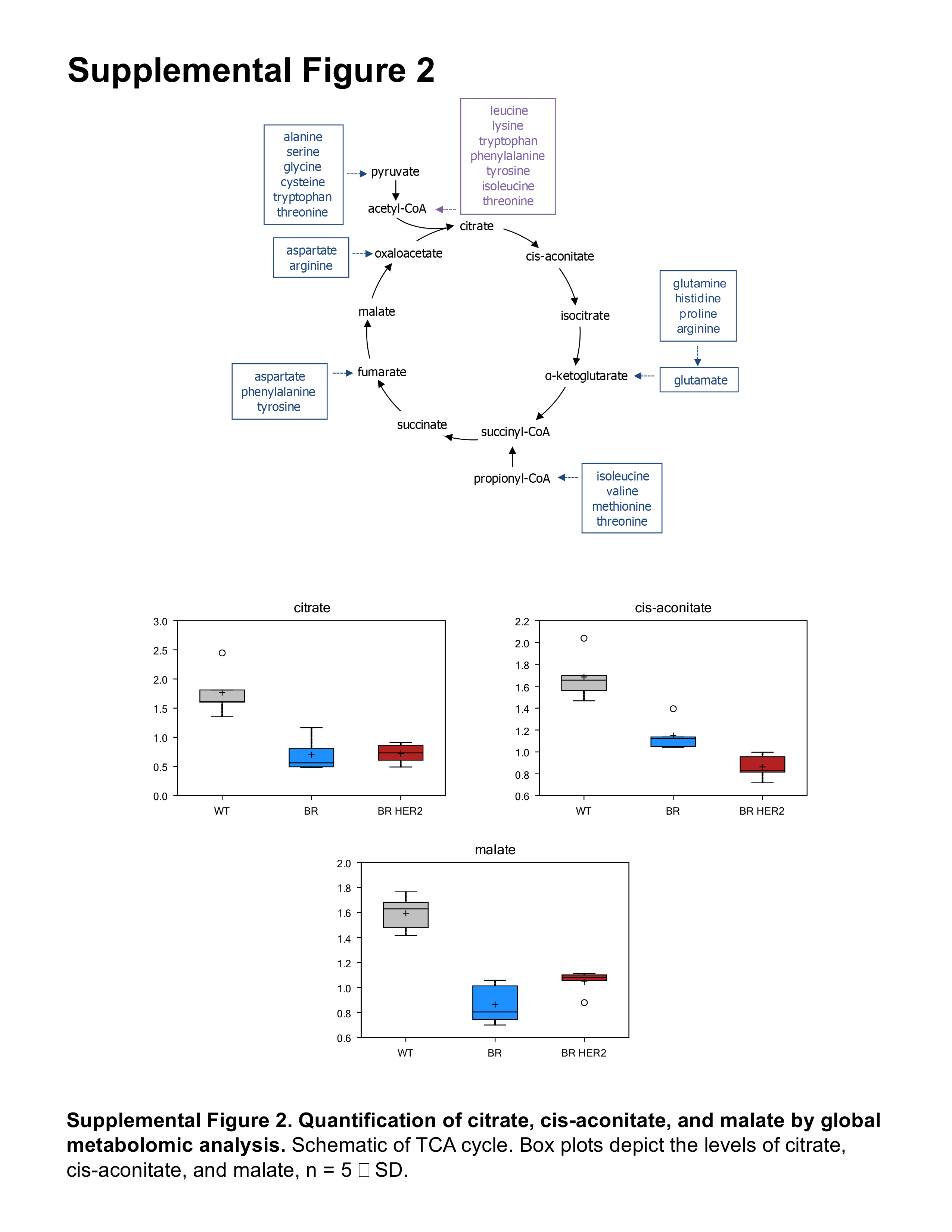

Supplement: Supplementary file 3 — Supporting Information [file CTM2-14-e1662-s001.jpg]

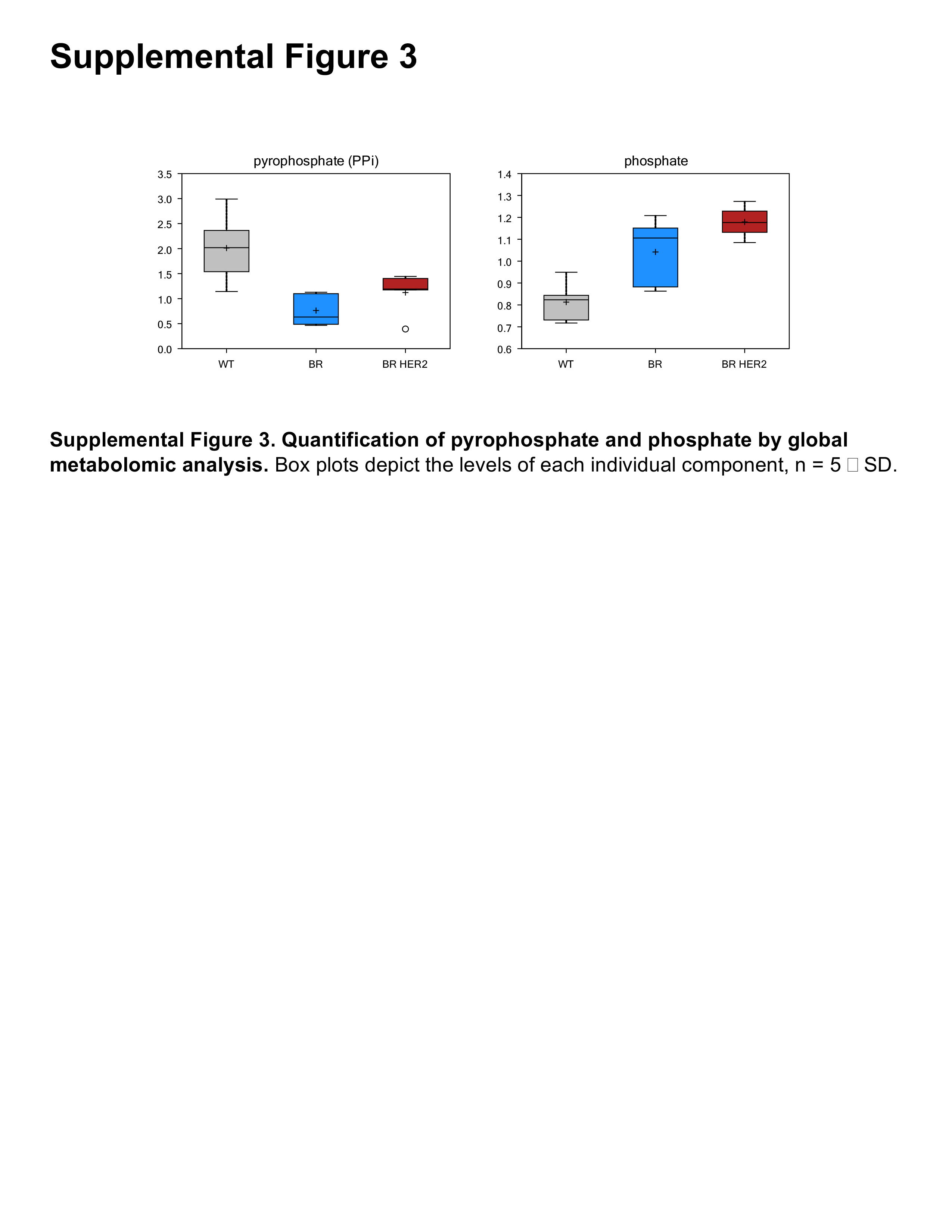

Supplement: Supplementary file 4 — Supporting Information [file CTM2-14-e1662-s004.jpg]

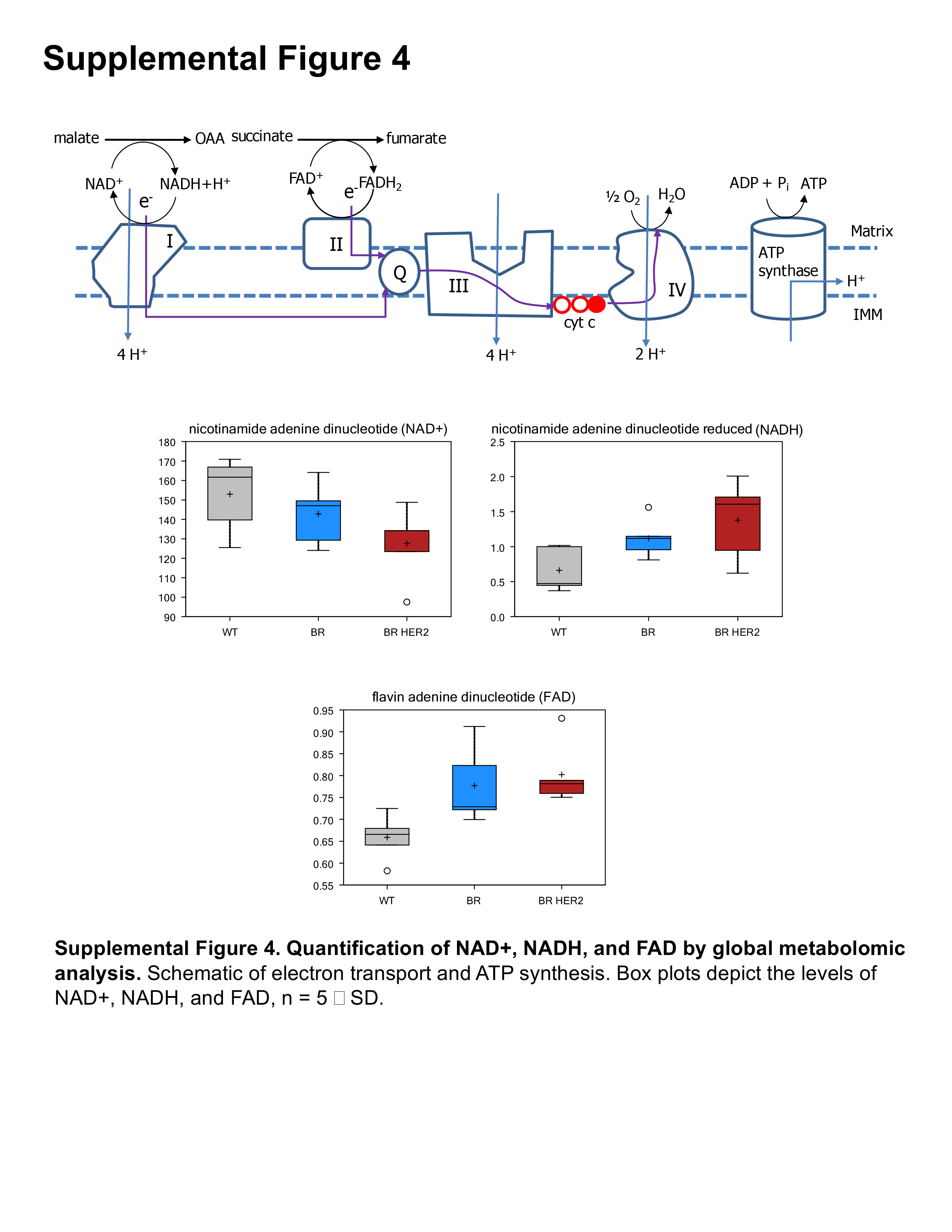

Supplement: Supplementary file 5 — Supporting Information [file CTM2-14-e1662-s005.jpg]

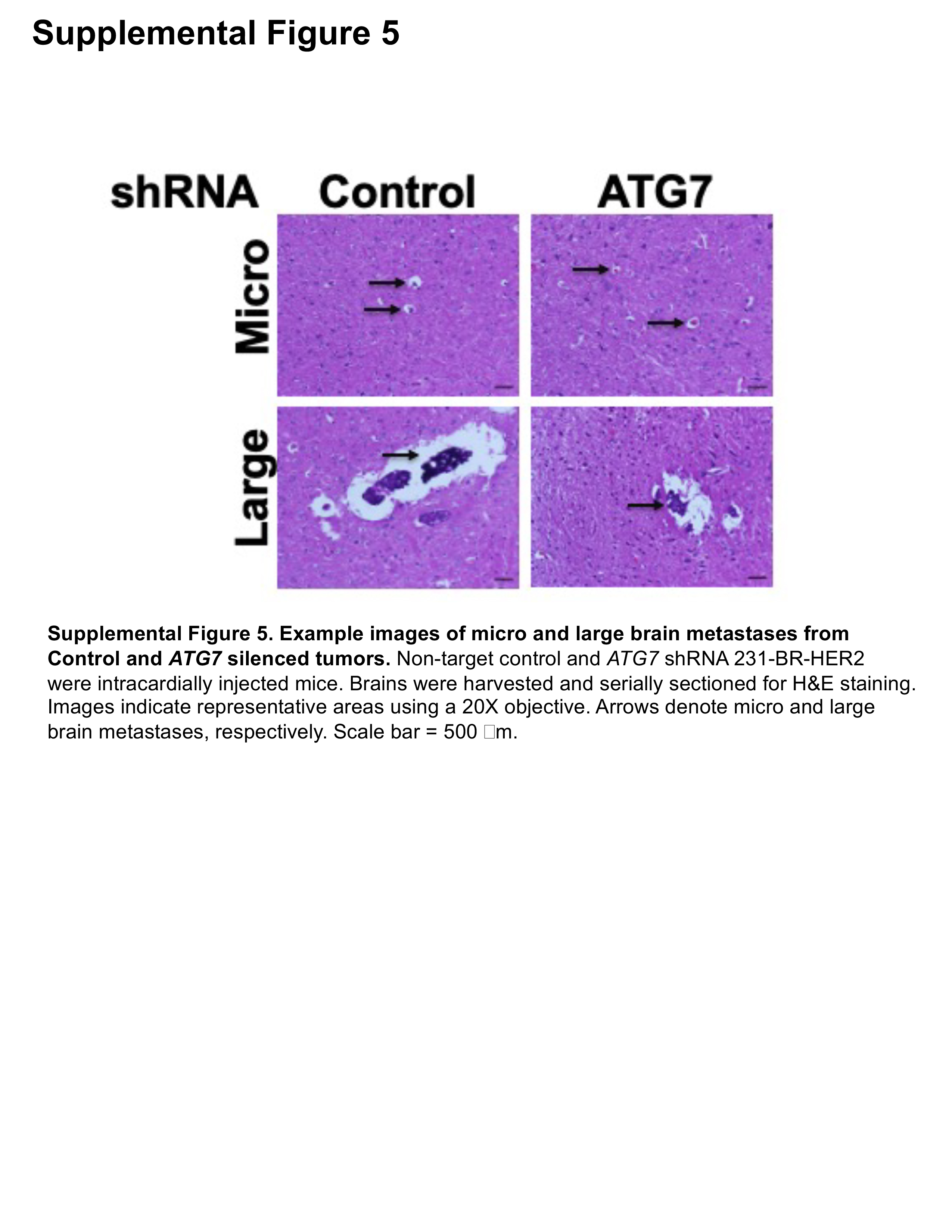

Supplement: Supplementary file 6 — Supporting Information [file CTM2-14-e1662-s002.jpg]
